# Dynamic change of lymphocytes associated with short-term prognosis in anti-MDA5-positive dermatomyositis with interstitial lung disease: a multicenter retrospective study

**DOI:** 10.1007/s10067-024-07110-3

**Published:** 2024-09-18

**Authors:** Yaqiong Tian, Ping He, Lijun Ren, Hongxia Xin, Bin Xi, Ruyi Zou, Qi Zhao, Xin Yan, Xiaohua Qiu, Yujuan Gao, Yin Liu, Min Cao, Hanyi Jiang, Bi Chen, Juan Chen, Hourong Cai

**Affiliations:** 1 Department of Respiratory and Critical Care Medicine, Nanjing Drum Tower Hospital, Clinical College of Nanjing Medical University, Nanjing, China; 2 Department of Respiratory and Critical Care Medicine, Affiliated Hospital of Medical School, Nanjing Drum Tower Hospital, Nanjing University, Nanjing, China; 3 Department of Respiratory and Critical Care Medicine, The Third People’s Hospital of Chengdu, Chengdu, China; 4 Department of Respiratory and Critical Care Medicine, Nanjing Drum Tower Hospital, Clinical College of Nanjing University of Chinese Medicine, Nanjing, China; 5 Department of Respiratory and Critical Care Medicine, General Hospital of Ningxia Medical University, Yinchuan, China; 6 Department of Respiratory and Critical Care Medicine, Affiliated Hospital of Xuzhou Medical University, Xuzhou, China

**Keywords:** Dermatomyositis, Interstitial lung disease, MDA5, Peripheral lymphocyte count

## Abstract

Lymphopenia is a unique manifestation of anti-MDA5 positive dermatomyositis with interstitial lung disease (MDA5 + DM-ILD). This study aimed to investigate the relationship between dynamic changes in peripheral lymphocytes and short-term prognosis in patients of MDA5 + DM-ILD. Two hundred sixty-three MDA5 + DM-ILD patients were divided into different groups according to lymphocyte count and death or survival within 1 month, then the differences in clinical features and outcomes were compared. Associations between lymphocytes and risk of death within 1 month were also investigated in different lymphocyte groups using Cox proportional hazard models. A generalized additive mixed model (GAMM) was established to analyze the dynamic changes of lymphocytes in the death 1-month group. Lymphocytes of the patients who died within 1 month were significantly lower than survivors by different lymphocyte grouping methods, and the total lymphocytes showed a gradually decreasing trend in non-survivors. And the difference between survivors and non-survivors was more obvious over time. The lowest tertile of baseline lymphocytes as a reference, the hazard ratios for death within 1 month in the highest tertile were 0.497 (95% CI 0.26–0.949, *P* for trend = 0.033) after adjustment for potential confounders. GAMM analysis found a mean daily decrease of lymphocytes (0.034 × 10^9/L) after admission in death 1-month patients. Low baseline lymphocytes and gradually declined lymphocytes are both associated with a high risk of death within 1 month. However dynamic changes in lymphocytes can better reflect the disease status and better predict the short-term prognosis than baseline lymphocytes in MDA5 + DM-ILD patients.

**Table:** 

**Key points** •*Low baseline lymphocytes and gradually decreased trend along time correlated with poor short-term prognosis in MDA5 + DM-ILD patients.* •*Dynamic changes of lymphocytes can better reflect the disease status and better predict the 1-month prognosis than baseline lymphocytes in MDA5 + DM-ILD patients.* •*Generalized additive mixed model (GAMM) analysis found that in 1-month non-survivors, peripheral blood lymphocytes decreased by 0.034 × 10^9/L per day, while the lymphocytes in survivors gradually increased.*

**Supplementary Information:**

The online version contains supplementary material available at 10.1007/s10067-024-07110-3.

## Introduction

Anti-melanoma differentiation-associated gene (MDA5) positive DM is a rare idiopathic inflammatory myopathy (IIM). In addition to skin and muscle involvement, it is most commonly involved in the lungs, manifested as interstitial lung disease (ILD), and the incidence can be as high as 50–100% [1]. Anti-MDA5 positive DM-ILD is usually presented as a rapidly progressive type [2], and the rapidly progressive interstitial lung disease (RP-ILD) is more likely to be MDA5 positive [3]. The disease often progresses rapidly and the treatment is difficult for these patients, despite receiving high-intensity systemic glucocorticoid and immunosuppressive therapy, the survival rate is still poor. Mortality is still as high as 40–50% within 6 months of diagnosis [4, 5] and most deaths occur in the early stages of the disease [6, 7]. MDA5-positive DM is more likely to implicate RP-ILD than anti-synthetase syndrome (ASS), and the survival rate of the patients is significantly lower than the patients with ASS [8]. Therefore, timely identification and prediction of short-term mortality risk in anti-MDA5 + DM-ILD patients is very important for guiding clinical treatment and improving patient prognosis.

Multiple previous studies have reported that peripheral blood lymphocytopenia is one of the characteristic clinical manifestations of anti-MDA5 + DM [9], and peripheral blood lymphocyte count can assist in judging the prognosis of patients [10]. Anti-MDA5 + DM patients are prone to implicate RP-ILD and also present with peripheral blood lymphocytopenia, and the decrease of peripheral blood lymphocytes in RP-ILD patients is associated with poor prognosis [11]. A previous study found that lymphocyte level was closely related to MDA5 + DM-associated lung interstitial lesions [9]. Baseline levels of lymphocytes could help early identification of high-risk patients with high mortality. The level of peripheral blood lymphocytes is promising as a clinical biomarker for the prognosis of MDA5 + DM patients [10]. However, the dynamic change of peripheral blood lymphocyte count in the disease course of anti-MDA5 + DM patients and its relationship with the prognosis of the patients have not been studied deeply.

In this study, both the baseline and dynamic changes of peripheral blood lymphocyte count in patients with anti-MDA5 + DM were analyzed in detail through different lymphocyte grouping and reliable statistical methods, demonstrating that dynamic changes of lymphocytes are closely related to the progression and short-term prognosis of the disease.

## Methods

### Study population

The study enrolled 263 effective patients of anti-MDA5 + DM-ILD who were diagnosed in four centers from 2017 to 2021, including The Affiliated Drum Tower Hospital of Nanjing University Medical School, Affiliated Hospital of Xuzhou Medical University, The Third People’s Hospital of Chengdu, and General Hospital of Ningxia Medical University. The criteria for diagnosis and classification are based on the 2017 EULAR/ACR classification criteria for IIMs [12]. Exclusion criteria include (1) age below 18 years old; (2) concurrent with other connective tissue diseases; (3) previous diagnosis of tumor; (4) incomplete clinical or laboratory data within 3 days after admission; (5) anti-MDA5 antibody positive combined with other myositis specific antibodies positive. This study was officially approved by the Ethics Committee of Nanjing Drum Tower Hospital, the Affiliated Hospital of Nanjing University Medical School (NO.: 2020–050-01).

## Definition of groups and variables

Patients who died within 1 month after the first hospitalization were defined as a “1-month non-survivor” group and patients who did not die within 1 month were defined as a “1-month survivor” group. Definition of peripheral blood Lymphocyte class: lymphocytes ≥ 1.10 × 10^9/L are normally defined as “class 1”, lymphocytes ≥ 0.55 × 10^9/L and < 1.10 × 10^9/L is a mild decrease defined as “class 2”, lymphocytes < 0.55 × 10^9/L is defined as “class 3” for a severe reduction [10]. In addition, patients also were divided into three groups according to tertile of baseline lymphocyte count. The lowest tertile was 0.490 × 10^9/L (*n* = 106), defined as “Lymphocyte group 0.490”, the range of lymphocytes was [0.08 to 0.60]; the median tertile was 0.785 × 10^9/L (*n* = 70), defined as “Lymphocyte group 0.785”, the range of lymphocytes was [0.61 to 0.90]; The highest tertile was 1.320 × 10^9/L (*n* = 87), defined as “Lymphocyte group 1.320”, the range of lymphocytes was [0.91 to 3.40]. Based on the first to 14th day after admission, the two adjacent days were combined into one group. For example, the first and second days were combined into one group and named T1-2 (Table 2). Variables covered in the study include age, gender, smoke, mechanic hand, heliotrope rash, Gottron's sign/papules, shawl sign, periungual erythema, Raynaud phenomenon, skin ulcers, muscle weakness, arthritis/arthralgia, RP-ILD, V sign, anti-nuclear antibody (ANA), rheumatoid factor (RF), Ro52, fever, lymphocyte class, lymphocyte count, duration, albumin(ALB), alanine aminotransferase(ALT), aspartate aminotransferase(AST), creatine kinase (CK), Lactate dehydrogenase (LDH), C-reactive protein (CRP), erythrocyte sedimentation rate (ESR), neutrophil to lymphocyte ratio (NLR), and white blood cell (WBC). RP-ILD was defined as dyspnea and hypoxemia within 3 months with the progression of imaging findings [13]. Duration was calculated from the onset of disease symptoms to diagnosis.

### Statistical analysis

All Statistical analyses were performed using IBM SPSS Statistics (Version 26.0) and R Studio (Version 3.6.3). *P* value of < 0.05 was considered statistically significant. Quantitative data are described as mean ± standard(SD) or median (first quartile, third quartile) according to data distribution. Qualitative data are presented as frequencies (percentages). Multiple comparisons were calculated by one-way ANOVA test, Welch’s *t*-test, Mann–Whitney *U* test, or Kruskal–Wallis test according to data distribution. The categorical variables were displayed as a percentage, and the chi-square test was used for qualitative data. The receiver operating characteristic curve (ROC curve) was conducted to analyze the predictive value of lymphocyte death within 1 month. For survival analysis, log-rank tests were applied for different patient subgroups. Smooth curve fitting, test for trend, and COX regression analysis by R Studio were utilized to evaluate the mortality risk with the Crude model and Adjusted model, adjusted variables in the adjusted model including age, gender, smoke, duration, mechanic hand, heliotrope rash, Gottron’s sign, V sign, shawl sign, periungual erythema, Raynaud phenomenon, skin ulcers, muscle weakness, arthritis, fever, ANA, RF, RO52, ALB, ALT, AST, CK, LDH, ESR, RP-ILD, CRP. Finally, we assessed the association between changed lymphocyte count (1–14th days) after admission and dead 1 month through the generalized additive mixed model (GAMM) with crude and fully adjusted models. GAMM is often applied in data obtained repeatedly like lymphocyte count in this study, especially when the data is repeated irregularly or with missing values [14, 15].

## Results

### Clinical characteristics of MDA5-positive ILD patients who died within 1 month

The total sample size was 263 patients, including 78 patients in the “1-month non-survivor” group and 185 patients in the “1-month survivor” group. The results showed that the lymphocyte count and Duration were significantly lower in 1-month non-survivors with statistical differences (Table 1). Age, ALB, AST, LDH, CRP, ESR, NLR, and WBC in the non-survivor group were significantly higher than those in the survivor group (Table 1). And between the two groups, gender, DM-associated skin vascular lesions such as Mechanic hand, heliotrope rash, Gottron’s sign, shawl sign, periungual erythema, Raynaud phenomenon, skin ulcer, and V sign were no significant statistical differences, nor were there differences in autoantibodies such as ANA, RF, and RO52 (Table 1). It is worth noting that the number of deaths within 1 month in RP-ILD patients was significantly higher than that in the non-RP-ILD group, the peripheral blood lymphocyte count in the “1-month non-survivor” group was lower, and the patients who died within 1 month were more distributed in the reduced lymphocyte group (class 2 and 3).

**Table 1:** Clinical characteristics of MDA5 + DM-ILD patients who died within 1 month

Variables	Total (*n* = 263)	1 month survivor (*n* = 185)	1 month non-survivor (*n* = 78)	*P* value
Demographic data				
Age, median, years	53.0[48.0,62.0]	51.0[46.0,58.0]	57.0[51.0,63.0]	< 0.001
Male, *n* (%)	176(66.9)	127(68.6)	49(62.8)	0.359
Smoke, *n* (%)	38(14.4)	21(11.4)	17(21.8)	0.028
Symptoms and disease status				
Fever, *n* (%)	138(52.5)	82(44.3)	56(71.8)	< 0.001
Duration, median, month	2.0[1.0,3.0]	2.0[1.0,4.0]	1.0[0.7,2.0]	< 0.001
RP-ILD, *n* (%)	121(46.0)	54(29.2)	67(85.9)	< 0.001
Cutaneous features				
Mechanic hand,n(%)	83(31.6)	54(29.2)	29(37.2)	0.203
Heliotrope rash, *n* (%)	114(43.3)	87(47.0)	27(34.6)	0.064
Gottron’s sign, *n* (%)	165(62.7)	118(63.8)	47(60.3)	0.589
Shawl sign, *n* (%)	32(12.2)	26(14.1)	6(7.7)	0.149
Periungual erythema, *n* (%)	33(12.5)	27(14.6)	6(7.7)	0.123
Raynaud phenomenon, *n* (%)	14(5.3)	10(5.4)	4(5.1)	0.927
Skin ulcers, *n* (%)	35(13.3)	26(14.1)	9(11.5)	0.583
V sign, *n* (%)	74(28.1)	58(31.4)	16(20.5)	0.074
Musculoskeletal features				
Arthritis, *n* (%)	93(35.4)	76(41.1)	17(21.8)	0.003
Muscle weakness, *n* (%)	70(26.6)	56(30.3)	14(17.9)	0.039
Laboratory findings				
Lymphocyte, median,10^9/L	0.7[0.5,1.1]	0.8[0.5,1.2]	0.6[0.5,0.9]	0.005
Lymphocyte class, *n* (%)				
1(≥ 1.10 × 10^9/L)	67(25.5)	55(29.7)	12(15.4)	0.046
2(0.55–1.10 × 10^9/L)	126(47.9)	85(46.0)	41(52.6)	
3(< 0.55 × 10^9/L)	70(26.6)	45(24.3)	25(32.0)	
ANA, positive,n(%)	110(41.8)	79(42.7)	31(39.7)	0.657
RF, positive, *n* (%)	23(8.7)	18(9.7)	5(6.4)	0.384
Ro52, positive, *n* (%)	203(77.2)	141(76.2)	62(79.5)	0.564
ALB, (mean ± SD), g/L	33.0 ± 4.2	33.9 ± 4.2	30.9 ± 3.3	< 0.001
ALT, median, units/L	53.9[30.6,82.7]	53.9[29.8,82.7]	52.7[33.3,84.6]	0.553
AST, median, units/L	47.3[32.0,73.3]	42.9[29.5,69.6]	60.9[39.8,80.0]	0.002
CK, median, units/L	55.0[30.0,133.0]	56.0[30.0,132.0]	51.0[27.0,152.0]	0.773
LDH, median, units/L	378.0[290.0,502.0]	343.0[265.0,454.0]	466.0[393.0,618.0]	< 0.001
CRP, median, mg/L	6.7[3.2,19.0]	5.1[2.7,12.3]	19.8[5.0,47.0]	< 0.001
ESR, median, mm/h	39.0[23.0,56.0]	33.0[20.0,51.0]	44.0[31.0,63.0]	< 0.001
NLR, median	5.9[3.5,10.0]	5.0[3.2,8.7]	8.7[5.4,13.3]	< 0.001
WBC, median, 10^9/L	5.9[4.3,7.9]	5.5[4.2,7.5]	6.5[4.8,9.5]	0.005

### The lymphocytes gradually decreased in 1 month non-survivors

The study analyzed the dynamic changes of peripheral blood lymphocyte count in the 1-month survivor and non-survivor group from the first day to the 14th day after admission and combined the adjacent 2 days into one group, including 7 groups in total. The results showed that the lymphocytes in 1-month non-survivors were significantly lower than in 1-month survivors in all seven groups from the first to 14th day, and along the time, the more obvious decrease of lymphocyte count was observed in “1-month non-survivor” group (Table 2). By comparing the baseline level of lymphocyte count and its short-term dynamic changes, we found that the total number of lymphocytes in 1-month non-survivors showed a gradual decrease trend with the progression of disease.

**Table 2:** The evolution of lymphocytes after admission between 1-month survivors and 1-month non survivors

Variables	Availabe value, *n*	Total (*n* = 263)	1 month survivor (*n* = 185)	1 month non-survivor (*n* = 78)	*P* value
Baseline lymphocyte,median		0.70[0.50,1.08]	0.80[0.54,1.20]	0.60[0.50,0.85]	0.005
T1-2, mean ± SD	231	0.69 ± 0.36	0.83 ± 0.40	0.52 ± 0.18	0.010
T3-4, median	207	0.70[0.50,1.00]	0.88[0.60,1.20]	0.62[0.38,0.70]	0.014
T5-6, median	178	0.80[0.41,1.10]	0.90[0.60,1.30]	0.50[0.38,0.80]	< 0.001
T7-8, median	184	0.70[0.46,1.10]	0.88[0.50,1.16]	0.60[0.39,0.81]	0.026
T9-10, median	215	0.65[0.48,1.20]	0.80[0.60,1.20]	0.32[0.21,0.60]	0.002
T11-12, median	203	0.72[0.40,1.24]	1.20[0.60,1.36]	0.43[0.30,0.58]	< 0.001
T13-14, median	178	0.68[0.39,1.30]	0.77[0.42,1.30]	0.40[0.22,0.64]	0.004

### Clinical characteristics of patients in different lymphocyte groups

According to the number of peripheral blood lymphocytes, they were divided into three classes [10]: class 1 with normal lymphocytes (*n* = 67), class 2 with mild lymphocyte reduction (*n* = 126), and class 3 with severe lymphocyte reduction (*n* = 70). Between the three groups, it was found that the higher RP-ILD ratio and inflammatory markers including LDH, CRP, ESR, and NLR and the shorter Duration were found in the low lymphocytes class, while there were no significant differences in DM-associated skin vascular lesions between three groups (Table 3). In terms of patient outcome, the lower the lymphocyte level, the higher 1 month, 6 months, and overall mortality rate. The mortality rate of class 2 and class 3 patients was significantly higher than that of the normal lymphocyte group (Table 3). Similar results were observed in another lymphocyte grouping method according to the tertile of the baseline lymphocytes (Supplementary Table S1), the specific group definition can be found in the methods part. These results suggested that the lower the level of peripheral blood lymphocytes, the higher the inflammatory factors, the higher the proportion of RP-ILD, and the higher the short-term mortality of patients.

**Table 3:** Clinical features of patients with different lymphocyte classes

Variables	Total (*n* = 263)	Lymphocyte class			*P* value
		1 (*n* = 67)	2 (*n* = 126)	3 (*n* = 70)	
Demographic data					
Age, mean ± SD,year	53.4 ± 11.0	50.7 ± 12.1	54.1 ± 10.4	54.8 ± 10.7	0.055
Male, *n*(%)	176(66.9)	51(76.1)	82(65.1)	43(61.4)	0.157
Smoke, *n*(%)	38(14.4)	4(5.9)	21(16.7)	13(18.6)	0.069
Symptoms and disease status					
Fever, *n* (%)	138(52.5)	32(47.7)	68(54.0)	38(54.3)	0.670
Duration, median, month	2.0[1.0,3.0]	2.0[1.0,5.0]	1.0[1.0,3.0]	1.0[0.7,3.0]	0.005
RP-ILD, *n* (%)	121(46.0)	18(26.9)	60(47.6)	43(61.4)	< 0.001
Cutaneous features					
Mechanic hand, *n* (%)	83(31.6)	20(29.9)	38(30.2)	25(35.7)	0.682
Heliotrope rash, *n* (%)	114(43.3)	31(46.3)	50(39.7)	33(47.1)	0.514
Gottron’s sign, *n* (%)	165(62.7)	44(65.7)	78(61.9)	43(61.4)	0.846
Shawl sign, *n* (%)	32(12.2)	11(16.4)	14(11.1)	7(10.0)	0.456
Periungual erythema, *n* (%)	33(12.5)	10(14.9)	14(11.1)	9(12.9)	0.745
Raynaud phenomenon, *n* (%)	14(5.3)	5(7.5)	7(5.6)	2(2.9)	0.480
Skin ulcers, *n* (%)	35(13.3)	12(17.9)	13(10.3)	10(14.3)	0.583
V sign, *n* (%)	74(28.1)	22(32.8)	35(27.8)	17(24.3)	0.534
Musculoskeletal features					
Muscle weakness, *n* (%)	70(26.6)	23(34.3)	27(21.4)	20(28.6)	0.141
Arthritis, *n* (%)	93(35.4)	29(43.3)	37(29.4)	27(38.6)	0.126
Laboratory findings					
ANA, positive, *n* (%)	110(41.8)	29(43.3)	47(37.3)	34(48.6)	0.297
RF,positive, *n* (%)	23(8.7)	7(10.4)	9(7.1)	7(10.0)	0.675
Ro52, positive, *n*(%)	203(77.2)	48(71.6)	99(78.6)	56(80.0)	0.444
ALB, mean ± SD, g/L	33.0 ± 4.2	34.9 ± 3.7	32.5 ± 4.0	31.9 ± 4.1	< 0.001
ALT, median, units/L	53.9[30.6,82.7]	57.0[32.0,92.1]	54.2[31.4,80.3]	47.8[27.4,83.2]	0.328
AST, median, units/L	47.3[32.0,73.3]	48.1[27.6,99.0]	46.6[33.0,67.7]	47.9[35.7,74.4]	0.839
CK, median, units/L	55.0[30.0,133.0]	55.0[27.0,153.0]	51.0[30.0,109.0]	65.0[30.0,134.0]	0.455
LDH, median, units/L	378.0[290.0,502.0]	336.0[264.0,439.0]	365.0[278.0,494.0]	439.0[329.0,613.0]	< 0.001
CRP, median, mg/L	6.7[3.2,19.0]	3.4[1.8,10.0]	7.5[3.9,21.2]	14.4[4.2,30.1]	< 0.001
ESR, median, mm/h	39.0[23.0,56.0]	36.0[23.0,44.0]	36.0[21.0,58.0]	41.0[29.0,63.0]	0.050
NLR, median	5.9[3.5,10.0]	3.2[2.2,5.3]	5.8[4.2,9.1]	11.5[6.0,18.9]	< 0.001
WBC, median, 10^9/L	5.9[4.3,7.9]	6.6[5.4,8.6]	5.5[4.3,7.9]	5.1[3.3,7.4]	< 0.001
Outcomes					
Overall outcome, *n* (%)	126(47.9)	21(31.3)	61(48.4)	44(62.9)	0.001
Death in 1 month, *n* (%)	78(29.7)	12(17.9)	41(32.5)	25(35.7)	0.046
Death in 3 months, *n* (%)	111(42.2)	21(31.3)	57(45.2)	33(47.1)	0.110
Death in 6 months, *n* (%)	117(44.5)	21(31.3)	59(46.8)	37(52.9)	0.031

### Association between baseline lymphocytes and death within 1 month

**Fig. 1:**
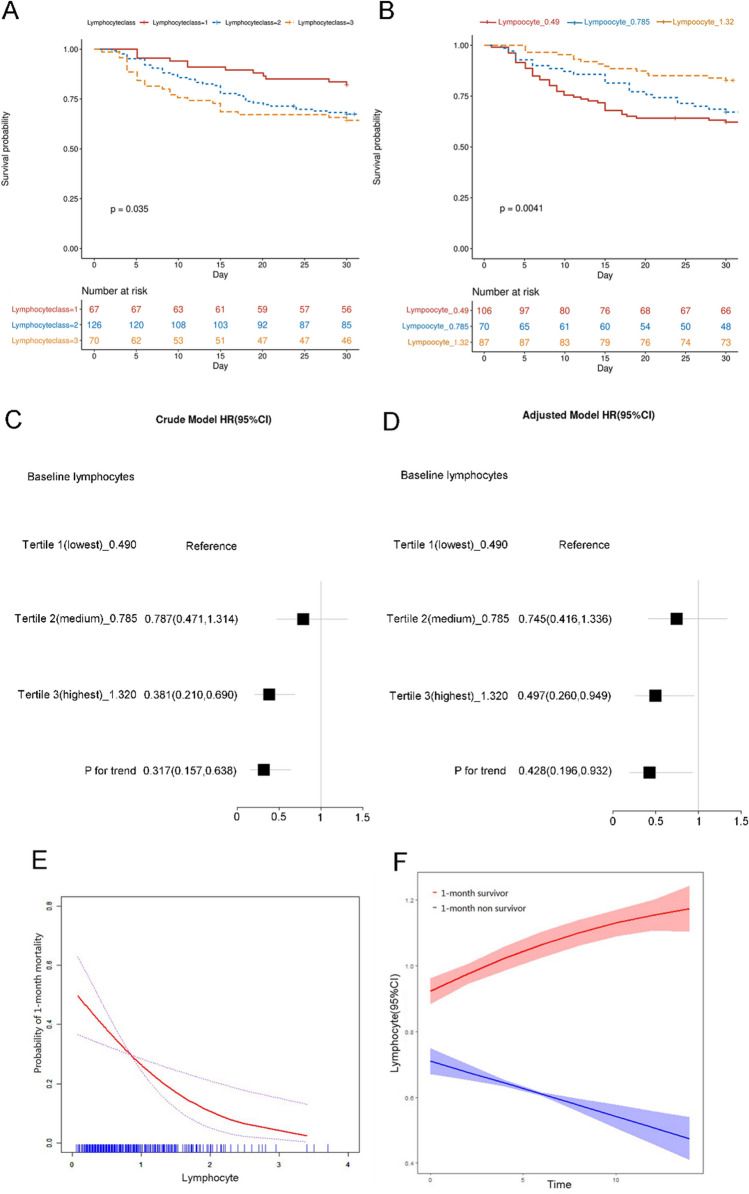
Correlation between changes of lymphocytes and risk of death within the short-term. **A**, **B** The survival rate of patients within one month in different lymphocyte groups was obtained by log-rank test. **C** The Crude model was constructed by test for trend analysis to analyze the correlation between lymphocyte and death within one month. **D** The Crude model was adjusted with confounding factors by combination with COX regression to further obtain Adjusted model and P for trend, the adjusted confounding factors included Gender, Duration, Smoke, Mechanic hand, Heliotrope rash, Gottron’s sign, V sign, Shawl sign, Periungual erythema, Raynaud phenomenon, Skin ulcer, Muscle weakness, Arthritis, Fever, ANA, RF, RO52, Age, ALT, AST, CK, LDH, ESR, RP-ILD, CRP. **E** The smooth curve fitting for the relationship between the baseline lymphocytes and the risk of 1-month mortality. **F** Association between changes of lymphocytes within 14 days in survivors and non-survivors

According to the ROC curve, the predictive value of peripheral blood lymphocytes in the patients who died within 1 month was obtained, the AUC was 0.61, the optimal cut-off value was 0.96, and the sensitivity was 37.7%, the specificity was 83.3% (Supplementary Figure S1). The Log Rank test analysis showed that the lower the number of lymphocytes group, the higher the mortality within 1 month with significant statistical differences. Consistent conclusions were reached in the two lymphocyte grouping methods, with *P* values of 0.035 and 0.004, respectively (Fig. 1A, B). The trend regression analysis (Fig. 1 C, D and Table 4) was conducted to analyze the relationship between the independent variable lymphocyte and the dependent variable “1 month non-survivor”, the adjusted model and P for trend were furtherly obtained by a combination of COX regression for confounding factors. The lowest tertile of lymphocytes group as reference, the risk of death within 1 month in the middle and the highest tertile group was 0.745 times (95% CI 0.416–1.336) and 0.497 times (95% CI 0.260–0.949), respectively (Fig. 1C, D). When the analysis was adjusted for potential confounders, the correlation was attenuated but remained significant (*P* for trend = 0.033) (Table 4). These results indicated that patients with lower lymphocyte numbers had a higher risk of death within 1 month.

**Table 4:** Risk of death within one month according to tertiles of baseline lymphocytes

Tertiles of lymphocytes	*N*	HR	95%CI	*P* value	*N* (adjusted)	HR (adjusted)	95%CI (adjusted)	*P* value
Tertile 1(lowest)_0.490	106				106			
Tertile 2(medium)_0.785	70	0.787	[0.471,1.314]	0.359	70	0.745	[0.416,1.336]	0.324
Tertile 3(highest)_1.320	87	0.381	[0.210,0.690]	0.001	87	0.497	[0.260,0.949]	0.034
P for trend				0.001				0.033

*HR* hazard odds ratio, *CI* confidence interval

### Dynamic decreasing lymphocytes correlated with death within 1 month

The smooth curve fitting showed that 1-month mortality increased with the reduction of lymphocyte (Fig. 1E). Furthermore, the changing trend of peripheral blood lymphocytes within 14 days showed that the lymphocytes in non-survivors showed a gradually decreasing trend whereas the lymphocytes in survivors were increasing, the difference of lymphocytes between two groups was more obvious with the extension of time (Fig. 1F). At 14th day, lymphocytes in non-survivors reduced about 0.38 × 10^9/L than survivors (P-value < 0.001) (Fig. 1F). Finally, a GAMM model (Table 5) was constructed to analyze the dynamic changes of peripheral blood lymphocyte count from 1 to 14 days in patients. By unadjusted model 1, model 2 (adjustment for clinical confounding variables), and model 3 (fully adjusted model), It was found that the average decrease of peripheral blood lymphocytes in the non-survivor group after admission was 0.034 × 10^9/L per day in the fully adjusted model (β =  − 0.0341, 95%CI (− 0.045, − 0.022), *P* value < 0.001) (Table 5). These results suggested that a gradual decrease in lymphocytes in patients is closely related to their 1-month mortality.

**Table 5:** Association between dynamic changes (1–14 days) of lymphocytes and death within 1 month in patients utilizing GAMM

One month mortality	Model 1		Model 2		Model 3	
	β(95%CI)	*P* value	β(95%CI)	*P* value	β(95%CI)	*P* value
Day	0.0163(0.0100,0.0225)	< 0.001	0.0165(0.0102,0.0227)	< 0.0001	0.0168(0.0105,0.0231)	< 0.001
Death	− 0.2236(− 0.3611, − 0.0861)	0.0015	− 0.1597(− 0.2966, − 0.0227)	0.0226	− 0.1579(− 0.2985, − 0.0173)	0.0281
Day × Death	− 0.0335(− 0.0451, − 0.0219)	< 0.001	− 0.0336(− 0.0452, − 0.0220)	< 0.0001	− 0.0341(− 0.0458, − 0.0224)	< 0.001

*Day*, the mean increase of lymphocytes daily in the survival group over time (1–14 days); *Death*, the mean difference of lymphocytes at admission between survivor group and non-survivor group; *Day* × *Death*, compared with survivor group, the mean decrease of lymphocytes per day in non-survivor group. *Model 1* unadjusted for any variables. *Model 2* adjusted for age, gender. *Model 3* adjusted for age, gender, duration, smoke, RP-ILD, mechanic hand, heliotrope rash, Gottron’s sign/papules, V sigh, shawl sign, periungual erythema, Raynaud phenomenon, skin ulcer, muscle weakness, arthritis, Ro52, fever, ANA, RF, ALB, ALT, AST, CK, LDH, CRP, ESR, WBC

## Discussion

Although previous studies have reported the correlation between peripheral blood lymphocytes and the prognosis of the disease [9, 10, 16], the dynamic changes of lymphocytes in MDA5-positive DM-ILD have not been fully analyzed. The advantages of this study include a relatively large sample size from multiple centers, and this study mainly focused on the relationship between dynamic changes of lymphocytes and the short-term prognosis of MDA5-positive DM-ILD. The mortality risk within 1 month was estimated based on different lymphocyte grouping methods, the lowest lymphocyte group had a higher mortality risk within 1 month compared with the highest group. By observing the dynamic changes of lymphocytes within 14 days after admission, it was found that the lymphocytes in 1-month non-survivors showed a gradually decreasing trend along the time, and a greater difference in lymphocytes was observed between survivors and non-survivors. Furthermore, by building the GAMM model, we found that the lymphocytes in 1-month non-survivors were reduced by about 0.034 × 10^9/L per day after admission. In this study, we applied several different statistical methods to prove our results, so they are accurate and reliable. GAMM model, a new statistical method, was first applied to dynamic changes of lymphocytes in this study since the changes of lymphocytes are repeated irregularly and with missing values.

Previous studies have reported that decreased peripheral blood lymphocytes are one of the clinical features of MDA5-positive DM patients [16, 17]. In this study, survival analysis revealed that the 3- and 6-month mortality were significantly higher in the severe lymphopenia group than in the mild lymphopenia and normal lymphocyte group, which were consistent with previous reports [10]. According to two different lymphocyte grouping methods in our study, the 1-month mortality was also significantly higher in the severe lymphopenia patients than in the mild lymphopenia and normal patients. Lymphocytopenia is one of the independent risk factors for RP-ILD in MDA5-positive DM-ILD patients, especially the reduction of CD3 + T cells, CD3 + CD4 + T cells, and CD3 + CD8 + T cells [8]. CD3 + CD8 + count ≤ 49.22 cell/μl and CD3‑CD19 + count ≤ 137.64 cell/μl were independent predictors of patient mortality risk [18]. In MDA5-positive DM-ILD patients, it was found that CD8 + T cells were reduced more significantly than CD4 + T cells in patients with RP-ILD, and the peripheral blood lymphocyte count was positively correlated with the degree of lung interstitial injury, but negatively correlated with the ratio of CD4:CD8 [9]. Specific lymphocyte subsets were not detailed in this study, thus the relationship between the dynamic changes of each lymphocyte subset and the prognosis of MDA5-positive DM-ILD patients can be explored in the future.

The results of this study also found that 121 of the 263 patients were RP-ILD, and 85.9% (67/78) of the patients died within 1 month were RP-ILD, while only 7.7% of the patients in the non-RP-ILD group died within 1 month, It is suggested that poor prognosis may be related to RP-ILD phenotype [2]. The short-term mortality of patients had no significant relationship with the skin vascular and joint lesions of patients [19]. ILD-associated blood biomarkers such as ferritin, Krebs von den Lungen-6 (KL-6), surfactant protein-D (SP-D) and cytokines can provide information on disease presence, activity, treatment response, and prognosis [20]. This study also found that ALB, LDH, CRP, ESR, NLR, and WBC were significantly higher in patients who died within 1 month, suggesting that MDA5-positive DM-ILD patients had higher levels of inflammation and the increase of inflammatory factors may be related to the short-term prognosis of patients [21].

At present, the treatment methods for MDA5-positive DM-ILD patients are limited. Current treatment regiments like systemic glucocorticoids and immunosuppressants such as tacrolimus and cyclophosphamide appear to have the highest survival rates in patients with RP-ILD, while plasma exchange can be added to refractory disease, thus early detection and treatment are extremely important [22]. A convenient, timely, and accurate predictor in clinical practice will help guide treatment. Lymphocyte count is a cheap-cost, widely available, easy-to-obtain marker of inflammation, which makes it more convenient for clinical use. This study can remind clinicians to pay great attention to the dynamic changes of peripheral blood lymphocytes and their importance to the reflection of disease status.

This study has some limitations, we only collected patients in China, and it is unclear whether our conclusions could be extended to other countries. And there are many factors affecting the level of lymphocyte count. In addition to the disease itself, immunosuppressant and glucocorticoid therapy could also affect lymphocyte count in clinical observation. Although previous studies reported that immunosuppressants have less influence on lymphocyte count and the lymphocytes could increase when the therapies were effective [9], these therapeutic factors may inevitably influence the results slightly. In conclusion, the low baseline level of peripheral blood lymphocytes and a gradually decreasing trend in the patients with MDA5-positive DM-ILD indicate a high mortality risk in the short term. And dynamic changes in lymphocytes can better reflect the disease status and prognosis than baseline lymphocytes. Clinicians need close monitoring of lymphocyte changes and take active treatment in time to improve the prognosis of patients.

## Supplementary Information

Below is the link to the electronic supplementary material.

Supplementary file1 (DOCX 438 KB)

## Data Availability

All data are available from the corresponding author on reasonable request.

## Abbreviations

MDA5: Melanoma differentiation-associated gene
MDA5 + DM-ILD: Anti-MDA5 positive dermatomyositis with interstitial lung disease
GAMM: Generalized additive mixed model
IIM: Idiopathic inflammatory myopathies
RP-ILD: Rapidly progressive interstitial lung disease
ASS: Anti-synthetase syndrome
ANA: Anti-nuclear antibody
RF: Rheumatoid factor
CK: Creatine kinase
LDH: Lactate dehydrogenase
CRP: C-reactive protein
ESR: Erythrocyte sedimentation rate
NLR: Neutrophil to lymphocyte ratio
WBC: White blood cell
KL-6: Krebs von den Lungen-6
SP-D: Surfactant protein-D

## References

[1] Motegi SI, Sekiguchi A, Toki S et al (2019) Clinical features and poor prognostic factors of anti-melanoma differentiation-associated gene 5 antibody-positive dermatomyositis with rapid progressive interstitial lung disease[J]. Eur J Dermatol 29(5):511–517 31617496 10.1684/ejd.2019.3634

[2] Betteridge Z, Tansley S, Shaddick G et al (2019) Frequency, mutual exclusivity and clinical associations of myositis autoantibodies in a combined European cohort of idiopathic inflammatory myopathy patients[J]. J Autoimmun 101:48–55 30992170 10.1016/j.jaut.2019.04.001 PMC6580360

[3] Xu L, You H, Wang L et al (2023) Identification of three different phenotypes in anti–melanoma differentiation–associated gene 5 antibody–positive dermatomyositis patients: implications for prediction of rapidly progressive interstitial lung disease. Arthritis Rheumatol 75(4):609–619 35849805 10.1002/art.42308

[4] Tsuji H, Nakashima R, Hosoo Y et al (2020) Multicenter prospective study of the efficacy and safety of combined immunosuppressive therapy with high-dose glucocorticoid, tacrolimus, and cyclophosphamide in interstitial lung diseases accompanied by anti-melanoma differentiation-associated gene 5-positive dermatomyositis. Arthritis Rheumatol 72(3):488–498 31524333 10.1002/art.41105

[5] You H, Wang L, Wang J et al (2023) Time-dependent changes in RPILD and mortality risk in anti-MDA5+ DM patients: a cohort study of 272 cases in China. Rheumatology 62(3):1216–1226 35961045 10.1093/rheumatology/keac450

[6] Li Y, Gao X, Li Y et al (2020) Predictors and mortality of rapidly progressive interstitial lung disease in patients with idiopathic inflammatory myopathy: a series of 474 patients[J]. Front Med (Lausanne) 7:363 32850886 10.3389/fmed.2020.00363 PMC7412929

[7] Chen Z, Cao M, Plana MN et al (2013) Utility of anti-melanoma differentiation-associated gene 5 antibody measurement in identifying patients with dermatomyositis and a high risk for developing rapidly progressive interstitial lung disease: a review of the literature and a meta-analysis[J]. Arthritis Care Res (Hoboken) 65(8):1316–24 23908005 10.1002/acr.21985

[8] Zuo Y, Ye L, Chen F et al (2022) Different multivariable risk factors for rapid progressive interstitial lung disease in anti-MDA5 positive dermatomyositis and anti-synthetase syndrome[J]. Front Immunol 13:845988 35320936 10.3389/fimmu.2022.845988 PMC8936070

[9] Huang W, Ren F, Luo L et al (2020) The characteristics of lymphocytes in patients positive for anti-MDA5 antibodies in interstitial lung disease[J]. Rheumatology (Oxford) 59(12):3886–3891 32535634 10.1093/rheumatology/keaa266

[10] Jin Q, Fu L, Yang H et al (2023) Peripheral lymphocyte count defines the clinical phenotypes and prognosis in patients with anti-MDA5-positive dermatomyositis[J]. J Intern Med 293(4):494–507 36682032 10.1111/joim.13607

[11] Li Y, Li Y, Wu J et al (2020) Predictors of poor outcome of anti-MDA5-associated rapidly progressive interstitial lung disease in a Chinese cohort with dermatomyositis[J]. J Immunol Res 2020:2024869 33299896 10.1155/2020/2024869 PMC7710415

[12] Lundberg IE, Tjärnlund A, Bottai M et al (2017) 2017 European League Against Rheumatism/American College of Rheumatology classification criteria for adult and juvenile idiopathic inflammatory myopathies and their major subgroups[J]. Ann Rheum Dis 76(12):1955–1964 29079590 10.1136/annrheumdis-2017-211468 PMC5736307

[13] Wu W, Guo L, Fu Y et al (2021) Interstitial lung disease in anti-MDA5 positive dermatomyositis[J]. Clin Rev Allergy Immunol 60(2):293–304 33405101 10.1007/s12016-020-08822-5

[14] Zhao X, Wang K, Zuo P et al (2020) Early decrease in blood platelet count is associated with poor prognosis in COVID-19 patients-indications for predictive, preventive, and personalized medical approach[J]. EPMA J 11(2):139–145 32419876 10.1007/s13167-020-00208-z PMC7224348

[15] Liu T, Wang B, Xiao S et al (2023) Correlation analysis between the static and the changed neutrophil-to-lymphocyte ratio and in-hospital mortality in critical patients with acute heart failure. Postgrad Med 135(1):50–57 36154549 10.1080/00325481.2022.2129177

[16] Lv X, Jin Y, Zhang D et al (2021) Low circulating monocytes is in parallel with lymphopenia which predicts poor outcome in anti-melanoma differentiation-associated gene 5 antibody-positive dermatomyositis-associated interstitial lung disease[J]. Front Med (Lausanne) 8:808875 35111785 10.3389/fmed.2021.808875 PMC8802832

[17] Wang H, Chen X, Du Y et al (2023) Mortality risk in patients with anti-MDA5 dermatomyositis is related to rapidly progressive interstitial lung disease and anti-Ro52 antibody[J]. Arthritis Res Ther 25(1):127 37488657 10.1186/s13075-023-03100-z PMC10367378

[18] Ren FP, Chen Q, Yao SS et al (2023) Characteristics and prognostic implications of peripheral blood lymphocyte subsets in patients with anti-MDA5 antibody positive dermatomyositis-interstitial lung disease[J]. BMC Pulm Med 23(1):411 37898737 10.1186/s12890-023-02706-y PMC10612305

[19] Allenbach Y, Uzunhan Y, Toquet S et al (2020) Different phenotypes in dermatomyositis associated with anti-MDA5 antibody: Study of 121 cases. Neurology 95(1):e70–e78 32487712 10.1212/WNL.0000000000009727 PMC7371381

[20] Li X, Liu Y, Cheng L et al (2022) Roles of biomarkers in anti-MDA5-positive dermatomyositis, associated interstitial lung disease, and rapidly progressive interstitial lung disease. J Clin Lab Anal 36(11):e24726 36221983 10.1002/jcla.24726 PMC9701872

[21] So J, So H, Wong VT et al (2022) Predictors of rapidly progressive interstitial lung disease and mortality in patients with autoantibodies against melanoma differentiation-associated protein 5 dermatomyositis. Rheumatology (Oxford) 61(11):4437–4444 35157042 10.1093/rheumatology/keac094

[22] McPherson M, Economidou S, Liampas A et al (2022) Management of MDA-5 antibody positive clinically amyopathic dermatomyositis associated interstitial lung disease: A systematic review[J]. Semin Arthritis Rheum 53:151959 35134633 10.1016/j.semarthrit.2022.151959

