# Supplementary material for: Dynamic change of lymphocytes associated with short-term prognosis in anti-MDA5-positive dermatomyositis with interstitial lung disease: a multicenter retrospective study
Source: Clin Rheumatol. 2024 Sep 18;43(11):3399–408. doi: 10.1007/s10067-024-07110-3 (PMC11489275; doi:10.1007/s10067-024-07110-3)

**Supplementary Table S1**. Clinical characteristics of the patients with MDA5+ DM-ILD grouping by tertile of the baseline lymphocyte count.


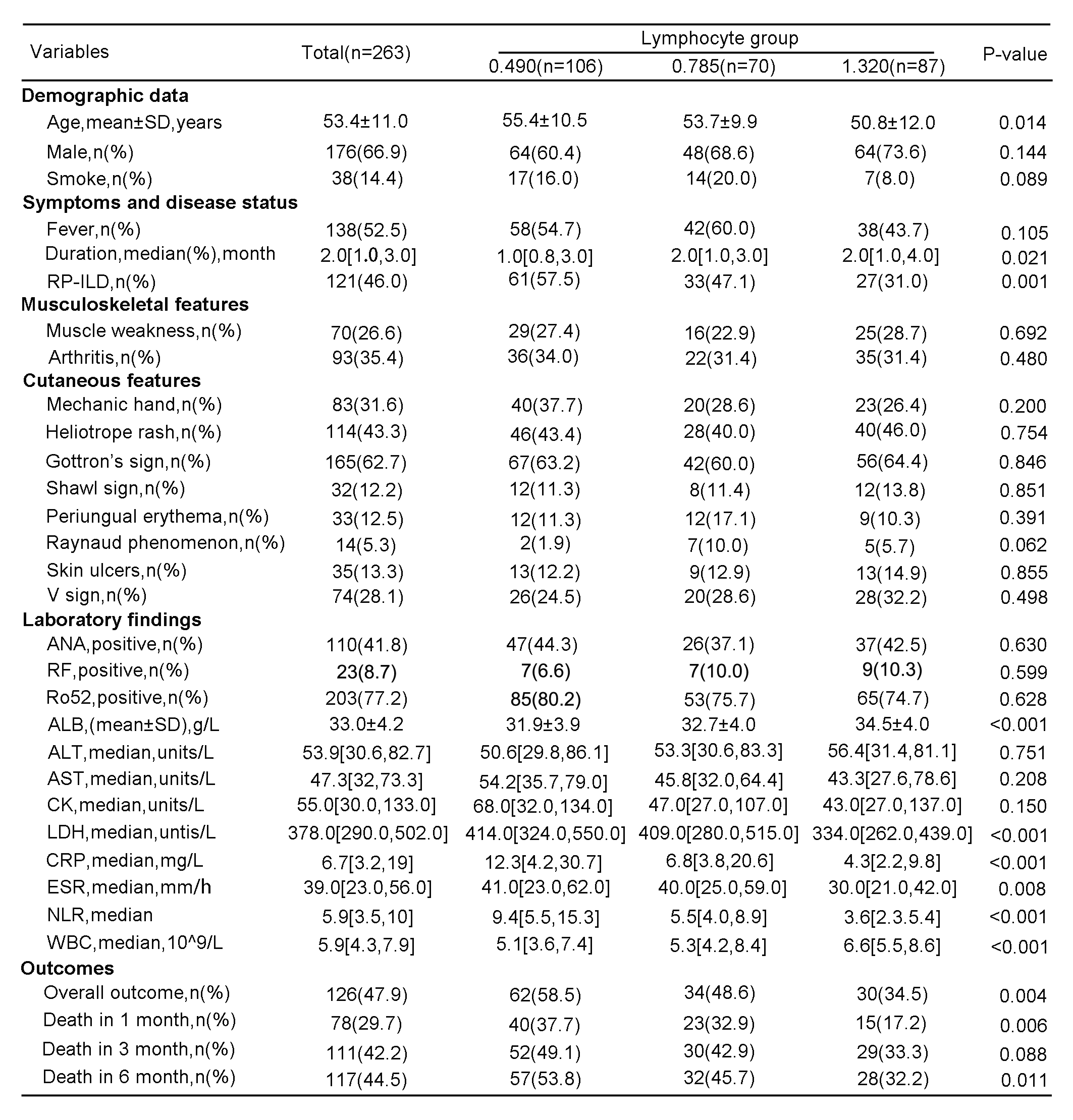


**Supplementary Fig S1**. The predictive value of baseline lymphocytes for risk of death within one month by ROC curve.


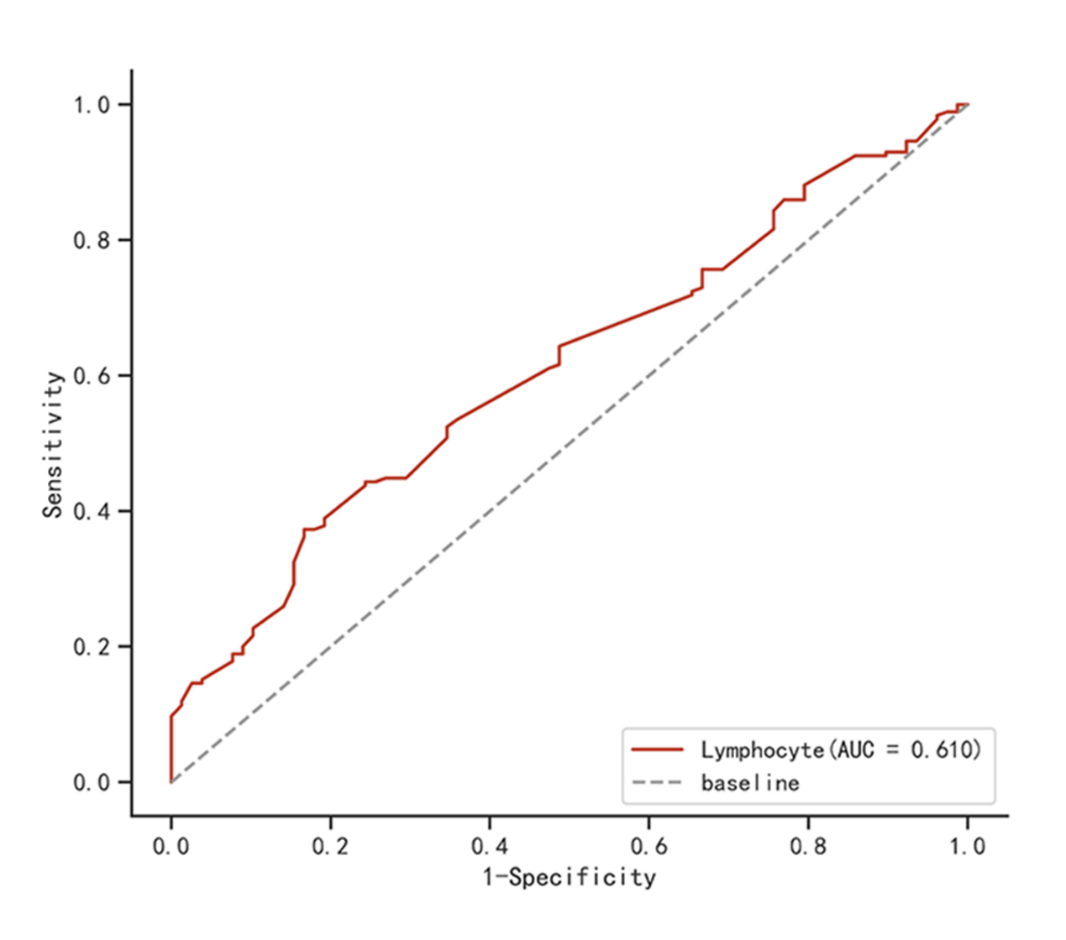

Supplement: Supplementary file 1 — Supplementary file1 (DOCX 438 KB) [file 10067_2024_7110_MOESM1_ESM.docx]
